# Supplementary material for: Mechanochemically Programmed, Oligomer‐Selective Amyloid Assembly via Axial Rotation
Source: Small. 2026 Aug 25;22(61):e75467. doi: 10.1002/smll.75467 (PMC13725412; doi:10.1002/smll.75467)
Supplement: Supplementary file 1 [file SMLL-22-e75467-s001.docx]

Supporting Information

**Mechanochemically Programmed, Oligomer-Selective Amyloid Assembly via Axial Rotation**

Seokbeom Roh^1,2,†^, Eunji Song^1,†^, Minwoo Bae^1^, Da Yeon Cheong^1,2^, Sechan Han^1^, Taeha Lee^1,3^,
Dain Kang^1^, Jae Eon Lee^1^, Seung Pil Pack^1^, Hyungbeen Lee^3,4^, Insu Park^5^, Gyudo Lee^1,2,3,4,6,*^

^1^ Department of Biotechnology and Bioinformatics, Korea University, Sejong 30019, Republic of Korea

^2^ Interdisciplinary Graduate Program for Artificial Intelligence Smart Convergence Technology, Korea University, Sejong 30019, Republic of Korea

^3^ Digital Healthcare Center, Sejong Institute for Business and Technology (SIBT), Korea University, Sejong 30019, Republic of Korea

^4^ Department of Digital Healthcare Engineering, Korea University, Sejong 30019, Republic of Korea

^5^ Department of Biomedical Engineering, Yonsei University, Wonju 26493, Republic of Korea

^6^ Biomedical Research Center, Korea University Guro Hospital, Seoul 08308, Republic of Korea

^†^ These authors contributed equally to this work.

*Corresponding author: Gyudo Lee (email: lkd0807@korea.ac.kr)

**Experimental Section**

**PeakForce Quantitative Nanomechanical Mapping**

PeakForce quantitative nanomechanical mapping (PeakForce-QNM) measurements were performed using a MultiMode VIII atomic force microscope (Bruker, USA) equipped with a J scanner and operated in PeakForce Tapping mode under ambient air conditions. RTESPA-525 probes (Bruker, USA) were used for both topographical imaging and nanomechanical characterization. The feedback gain and PeakForce setpoint were set to 30 and 10 nN, respectively. The PeakForce amplitude and frequency were set to 50 nm and 2 kHz, respectively.

Before each measurement, the AFM probe was calibrated using the relative calibration method to ensure consistent determination of the elastic modulus. Briefly, the deflection sensitivity was calibrated using a sapphire reference sample, whereas a polystyrene (PS) reference sample supplied by Bruker was used for modulus calibration. The PS calibration standard had a Young’s modulus within the nominal range of 1–20 GPa. The DMT modulus was obtained by fitting the force–distance curves acquired at each image pixel using the Derjaguin–Muller–Toporov contact-mechanics model implemented in the Bruker analysis software. Representative height and DMT modulus maps were acquired from independently selected regions of each sample. Image processing and quantitative analysis of the height and DMT modulus distributions were performed using MountainsSPIP software (version 9; Digital Surf, France).

**Protein Gel Electrophoresis**

Tris–glycine sodium dodecyl sulfate–polyacrylamide gel electrophoresis (SDS-PAGE) was performed under reducing conditions to analyze the protein profiles of HEWL samples. Fresh monomeric HEWL and samples subjected to axial rotation at 0, 350, 600, and 900 RPM were diluted to 2 mg mL⁻¹ and mixed with 5× SDS-PAGE loading buffer (CBS002, LPS Solution, Republic of Korea). The samples were analyzed without heat treatment. Proteins were separated using Tris–HCl gels consisting of a 15% resolving gel and a 5% stacking gel in Tris–glycine–SDS running buffer. Protein bands were visualized by Coomassie Brilliant Blue G-250 staining.

**Size-exclusion chromatography (SEC)**

Hen egg-white lysozyme (HEWL) samples, including monomeric HEWL and samples treated at 0, 350, 600, and 900 RPM, were analyzed by fast protein liquid chromatography (FPLC). Size-exclusion chromatography was performed using a Superdex™ 75 Increase 10/300 GL column (Cytiva, Uppsala, Sweden; Cat. No. 29148721) mounted on an ÄKTA™ purifier UPC 100 FPLC system (Cytiva, Uppsala, Sweden; Cat. No. 29011324), and chromatographic data were acquired and analyzed using UNICORN™ software version 5.31 (Cytiva, Uppsala, Sweden). Prior to sample injection, the column was washed with 2 column volumes (CV) of Ultra Pure Water (BIOSESANG, Inc., Republic of Korea; Cat. No. WR4006-100-00), followed by equilibration with 1 CV of running buffer consisting of 100 mM glycine, adjusted to pH 2.0 with HCl. HEWL samples were diluted in 50 mM NaCl buffer adjusted to pH 2.0 with HCl, and 1 mg of each sample was injected onto the column. Separation was performed at a flow rate of 1.0 mL/min for 35 min, and elution profiles were monitored by UV absorbance at 280 nm.

**Note S1. Calculation of RPM-Dependent Hydrodynamic Parameters**

To compare the RPM-dependent scaling of the mechanical inputs generated by axial rotation, representative hydrodynamic parameters were calculated using the measured cylindrical inner diameter of the Hyundai Micro 50 mL conical tube. The inner diameter was 27.0 mm, corresponding to a cylindrical inner radius of *R* = 13.5 mm. For a rotational speed *N*, the angular velocity was calculated as

$$\omega=\frac{2\pi N}{60},$$

where *N* is expressed in revolutions per minute. The centrifugal acceleration and corresponding relative centrifugal force were calculated as

$$a_{c}=\omega^{2}R$$

and

$$RCF=\frac{a_{c}}{g},$$

respectively, where *g* = 9.80665 m·$s^{-2}$.

To describe the RPM-dependent wall-speed contribution, a nominal shear-rate scale was defined as

$$\dot{\gamma}_{\mathrm{nom}}=\frac{\omega R}{\delta},$$

where *δ* = 1.00 mm was fixed as an assumed characteristic velocity-gradient length. This value was introduced solely to enable comparison among rotational conditions and was not experimentally measured; therefore, it should not be interpreted as the actual hydrodynamic boundary-layer thickness. The corresponding nominal viscous-stress scale was calculated as

$$\tau_{\mathrm{nom}}=\eta\dot{\gamma}_{\mathrm{nom}}.$$

A nominal dynamic viscosity of *η* = 0.4908 mPa·s was estimated using the first-order relation

$$\eta\approx\eta_{w}(1+[\eta]c),$$

with the viscosity of water at 60 °C, $\eta_{w}$ = 0.46604 mPa·s, an aqueous native-like HEWL intrinsic viscosity of $[\eta]$ = 2.66 mL/g, and a HEWL concentration of *c* = 0.020 g/mL. Because the available HEWL viscosity data were obtained under conditions different from the present pH 2, 60 °C, aggregation-prone system, this viscosity was used as a literature-informed fixed scaling parameter rather than as a directly measured sample viscosity [1, 2].

The calculations do not explicitly resolve the conical bottom of the tube, RPM-dependent liquid-film thickness and liquid-contact radius, free-surface deformation, or partial liquid co-rotation. In particular, the 1 mL sample initially occupies the conical lower region, where the local radius is smaller than the cylindrical radius used in the calculations. As RPM increases, the liquid climbs the tube wall, and both the effective contact radius and liquid-film geometry change. The calculated *a_c_* and *RCF* values therefore represent values evaluated at the cylindrical inner radius, while $\dot{\gamma}_{\mathrm{nom}}$ and $\tau_{\mathrm{nom}}$ are nominal wall-speed-based scales rather than measurements of the local wall shear rate or viscous stress.

Under these fixed assumptions, *a_c_* and *RCF* increase with RPM squared, whereas $\dot{\gamma}_{\mathrm{nom}}$ and $\tau_{\mathrm{nom}}$ increase linearly with RPM (Figure S1). These parameters are therefore used to visualize the relative scaling of the coupled centrifugal and wall-associated mechanical inputs. They are not intended to isolate the contribution of an individual hydrodynamic parameter or to assign a specific microscopic amyloid-assembly process to any single calculated value.

**Figure S1. RPM-dependent scaling of calculated hydrodynamic parameters during axial rotation.**

(a) Calculated centrifugal acceleration, *a_c_*; (b) Calculated relative centrifugal force, *RCF*; (c) nominal shear-rate scale, $\dot{\gamma}_{\mathrm{nom}}$; and (d) nominal viscous-stress scale, $\tau_{\mathrm{nom}}$. The parameters were calculated using *R* = 13.5 mm, corresponding to the cylindrical inner radius of the 50 mL conical tube, an assumed characteristic velocity-gradient length of *δ* = 1.00 mm, and a nominal dynamic viscosity of *η* = 0.4908 mPa·s. The calculations do not explicitly account for the smaller local radius of the conical bottom, RPM-dependent liquid-film geometry, free-surface deformation, or liquid co-rotation. These parameters illustrate the relative scaling of centrifugal and wall-associated mechanical inputs with increasing RPM rather than direct measurements of the local hydrodynamic field.

**Figure S2. Volume- and RPM-dependent deformation of the air–water interface.**

Representative images of the liquid interface at (a) 1 mL, (b) 2 mL, (c) 5 mL across increasing rotational speeds. Progressive wall climbing and interfacial tilting are observed with increasing RPM.

**Figure S3. Surface temperature stability of the rotating tube across RPM conditions.**

(a) Schematic of the axial rotation system and representative infrared thermal image used for surface temperature analysis. The conical tube was mounted on the axial rotator positioned in front of heat blocks, and thermal distribution was monitored using an infrared thermal imaging camera. The thermal map shows the spatial temperature profile of the tube surface under rotational conditions, with the color scale indicating temperature (30–90°C). (b) Quantitative analysis of tube surface temperature as a function of rotational speed (0–1200 RPM). Temperature values were extracted from a defined region of interest along the central tube wall in the infrared thermal images. Data are presented as mean ± SD (n = 4).

**Figure S4. Protein concentration analysis by bicinchoninic acid (BCA) assay.**

Protein concentration of samples prepared under control and various rotational speeds (0–1200 RPM) measured using the BCA assay. Concentrations are expressed in µg/mL. Data are presented as mean ± SD (n = 3). No statistically significant differences were observed among conditions (ns).

**Figure S5. Circular dichroism (CD) spectra of amyloid samples prepared under different rotational conditions.**

Far-UV CD spectra (190–240 nm) of monomer (Mono) and samples generated at 0, 350, 600, and 900 RPM. Samples were diluted in pH 2 DW, and the corresponding buffer spectrum was subtracted.

**Figure S6. High-resolution AFM analysis of particulate assemblies formed at high rotational speeds.**

Representative atomic force microscopy (AFM) height images of samples prepared at (a) 600 RPM and (b) 900 RPM after incubation at 60 °C for 3 days. Dashed boxes indicate regions selected for magnified views shown below. White arrows denote representative particulate assemblies.

**Figure S7. PeakForce quantitative nanomechanical mapping of HEWL amyloid assemblies generated at 600 and 900 RPM.**

(a) Representative AFM height images of the 600 and 900 RPM assemblies. Colored boxes indicate the regions magnified in (b). (b) Magnified height images and (c) corresponding DMT modulus maps. (d, e) Violin plots of particle height and DMT modulus, respectively. Particle height did not differ significantly between the two conditions, whereas the 900 RPM assemblies exhibited a significantly higher DMT modulus than the 600 RPM assemblies. Individual points represent measured particles (n = 30 particles per condition). Statistical significance was evaluated using an unpaired two-tailed t-test. Scale bars: 1 μm in (a) and 250 nm in (b) and (c). ns, not significant; *p < 0.05.

**Figure S8. SDS–PAGE analysis of HEWL samples prepared under different axial-rotation conditions.**

Coomassie Brilliant Blue-stained SDS–PAGE gel of freshly prepared HEWL monomer controls and samples incubated at 60 °C for 3 days under static or axial-rotation conditions. Lanes from left to right correspond to the protein ladder, two independently loaded HEWL monomer controls, and samples prepared at 0, 350, 600, and 900 RPM. The intact HEWL monomer band was detected at approximately 14 kDa. Incubated samples additionally exhibited lower-molecular-weight bands below the monomer position, with fewer and/or weaker bands in the 600 and 900 RPM samples than in the 0 and 350 RPM samples.

**Figure S9. Size-exclusion chromatography (SEC) profiles of HEWL samples prepared under different axial-rotation conditions.**

SEC profiles of freshly prepared HEWL monomer and samples incubated at 60 °C for 3 days at 0, 350, 600, and 900 RPM. (a) Full elution profiles from 10 to 35 mL. (b) Expanded view of the principal protein-elution region from 20 to 26 mL; the blue dashed line indicates the elution position of freshly prepared monomeric HEWL. The chromatograms are interpreted as comparative elution-volume distributions rather than absolute molecular-weight assignments.

**Figure S10. AFM characterization of aggregates formed in the seeding experiments (corresponding to Figure 4).**

Representative atomic force microscopy (AFM) height images of samples collected after 3 days of incubation under three conditions: (a) monomer only, (b) monomer mixed 1:1 with oligomer-dominant assemblies generated at 600 RPM, and (c) monomer mixed 1:1 with oligomer- and fibril-containing assemblies generated at 350 RPM.

**Figure S11. Live/dead assay of SH-SY5Y cells treated with RPM-defined HEWL assemblies at 7 μM.**

HEWL samples were prepared at 60 °C for 3 days under different rotational speeds (Monomer, 0, 350, 600, and 900 RPM) and applied to SH-SY5Y cells at 7 μM. (a) Representative fluorescence images of live/dead staining. Live cells are shown in green and dead cells in red. Scale bars: 200 µm. (b) Quantification of live and dead cell populations expressed as percentages of total cells for each condition.

**Figure S12. High-magnification fluorescence images of cytoskeletal and nuclear staining (corresponding to Figure 6).**

Representative confocal fluorescence images of SH-SY5Y cells treated with RPM-defined HEWL assemblies (Monomer, 0, 350, 600, and 900 RPM; 60 °C, 3 days). F-actin was stained with Phalloidin–TRITC (red), and nuclei were stained with DAPI (blue). Merged images are shown. Scale bars: 20 µm.

**Table S1. Quantitative secondary-structure composition of HEWL assemblies generated under different axial-rotation conditions, as estimated from far-UV circular dichroism spectra.** Values represent the estimated fractions (%) of α-helix, β-sheet, β-turn, and random-coil structures obtained using the Protein Secondary Structure Estimation module.

**References**

1.	Huber, M. L.; Perkins, R. A.; Laesecke, A.; Friend, D. G.; Sengers, J. V.; Assael, M. J.; Metaxa, I. N.; Vogel, E.; Mareš, R.; Miyagawa, K., *Journal of Physical and Chemical Reference Data* **2009,** *38* (2), 101-125. DOI 10.1063/1.3088050.

2.	Monkos, K., *Biochimica et Biophysica Acta (BBA) - Protein Structure and Molecular Enzymology* **1997,** *1339* (2), 304-310. DOI <https://doi.org/10.1016/S0167-4838(97)00013-7>.
